# Low-Dose Prednisone Treatment for IVIG-Resistant Kawasaki Disease with Severe Arthritis and Joint Effusion in Two 3-Year-Old Children

**DOI:** 10.1155/2021/6618346

**Published:** 2021-04-02

**Authors:** Lingling Fan, Huimin Lv, Shujuan Jiang, Daogang Qin

**Affiliations:** Department of Paediatrics, Liaocheng People's Hospital, Liaocheng, Shandong, China

## Abstract

Kawasaki disease (KD) is a global disease in children. The etiology and pathogenesis are unknown. Complications vary among patients. Fever can persist in some after immune globulin (IVIG) administration, termed IVIG-resistant KD. Here, we report two cases of IVIG-resistant KD with severe arthritis. The diagnosis of arthritis was confirmed by magnetic resonance imaging (MRI) showing joint effusion. Remarkably, fever and joint pain had not receded after the second dose of IVIG. To further manage the symptoms, we prescribed low-dose oral prednisone with success. Both fever and joint pain were diminished. We ponder that the low-dose prednisone might be an option to treat IVIG-resistant KD with severe arthritis.

## 1. Introduction

Kawasaki disease- (KD-) related arthritis has been reported to occur in 2.3–31% of children with KD [1]. The detailed study on this subject is lacking, and pathogenesis is unknown. Approximately 10% to 20% of patients with KD are IVIG resistant [2]. The incidence of IVIG-resistant KD combined with severe arthritis is infrequent, and there is no standardized treatment. We report two such cases with successful treatment in an attempt to offer a hint that may contribute to establishing a standardized treatment strategy in future. Considering these two cases having multiple symptoms, we carefully characterized the diagnosis. The main differential diagnosis is systemic-onset juvenile idiopathic arthritis (SOJIA), which had been excluded by negative antinuclear antibodies (ANA) in these two patients. Particularly, they had periungual desquamation towards the end of their illness course, a unique feature of KD. Lastly, they presented persistent rash, which is uncommon in SOJIA, but can be seen in KD quite often. Once the final diagnoses were made, we started IVIG followed by administration of low-dose prednisone till no symptom was seen.

## 2. Case Presentation

### 2.1. Case One

A 3-year-old Chinese girl was admitted with a chief complaint of fever (>40.0°C) and rashes for eight days. The rashes covered the entire body, and there was no itching. She had occasional nonproductive cough. Four days after fever, she gradually developed pain in her bilateral legs and fingers. She had difficulty walking and gripping objects due to pain. She was given oral cefoxime and azithromycin for three days without any improvement. Three days before admission, she was diagnosed with classic KD at a local hospital. Antibiotics were discontinued, and intravenous immunoglobulin (IVIG, 2 g/kg) and aspirin (30 mg/kg/day) were started. However, here, fever persisted and pain in legs and fingers was more pronounced. On examination, she was febrile (38.6°C) and tachycardic (146 beats/min). Symptoms were resolved by antipyretics only temporally. She was bed-bounded and had pale skin and ill appearance. Rashes covered most of her skin surface. Multiple cervical lymph nodes were palpable. Eyeballs were pinkish red with clear discharge (nonpurulent conjunctivitis). Lips were dry and cracked. The tongue was red and bumpy and strawberry looking. Pharyngeal erythema without exudate was evident. No abnormality was observed in the respiratory, gastrointestinal, and cardiac systemic review. Metacarpophalangeal (MCP) joints had noticeable redness and swelling and tender to touch. She was able to move her hips, knees, and ankles freely at a supine position but unable to do so when standing due to pain. Her palms and soles turned red and swollen.

**Figure 1:**
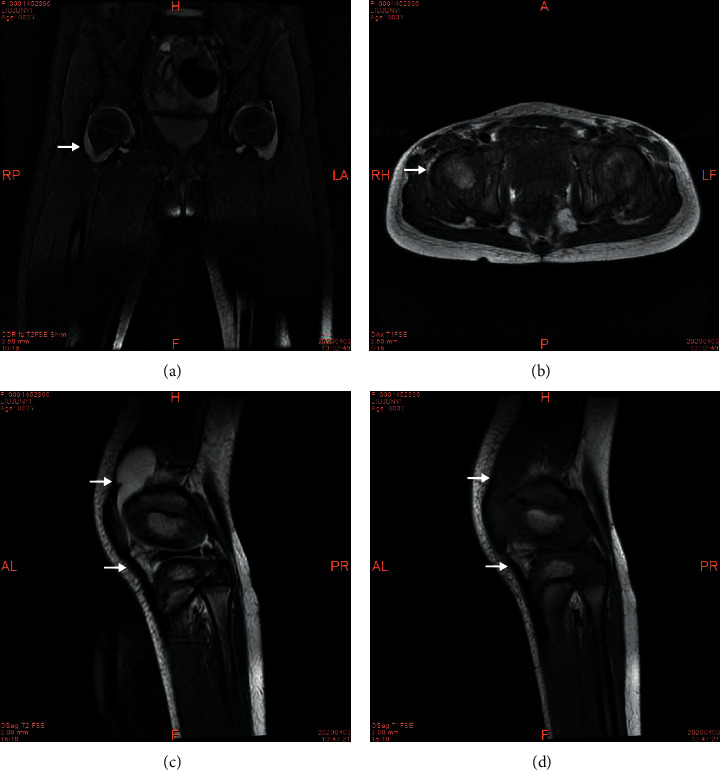
T1-weighed, T2-weighed, and Fat-suppressed T2-weighted imaging in the acute phase of Kawasaki disease. There were a long T1 signal and long T2 signal in the bilateral hips (a), (b), knees, and bursae suprapatellaris (c), (d), which indicated arthritis with effusion.

On laboratory investigation, she had elevated white cell count (WCC) at 18.17 × 10^9^/L (reference rage: 3.5–9.5 × 10^9^/L), decreased haemoglobin at 88 g/L (reference range: 120–140 g/L), and normal platelet count at 377 × 10^9^/L (reference range: 100–300 × 10^9^/L). On the comprehensive metabolic panel (CMP), She had a lower level of albumin at 22 g/L (reference range: 40–55 g/L) and normal electrolytes, calcium, magnesium, and phosphate. On the inflammatory markers test, she had elevated c-reactive protein (CRP) at over 200 mg/L (reference range: 0–10 mg/L), erythrocyte sedimentation rate (ESR) at 111 mm/h (reference range: 0–20 mm/h), and interleukin-6 (IL-6) at 303.8 pg/mL (reference range: ≤5.4 pg/mL). On lumbar puncture (LP), her cerebrospinal fluid pressure (CSFP) was 190 mmH_2_O (reference range: 80–180 mmH_2_O]. The nucleated cell from the cerebrospinal fluid (CSF) increased in number with 90 × 10^6^/L (reference rage: 0–8 × 10^6^/L). Other lab findings were normal brain natriuretic peptide (BNP), negative anti-*Streptococcus* “O” antibody, negative blood culture, and negative detection for antibodies against Rheumatoid Factor (RF), ANA, ds-DNA, Sm, SS-A, SS-B, U1RNP, RO-52, SCL-70, and PM-Scl. The echocardiogram revealed that the coronary arteries were normal. The left main coronary artery (LMCA) was 2.3 mm (Z score = 0.65); right coronary artery (RCA) was 1.9 mm, (Z score = 0.32). A standard 12-lead electrocardiogram (ECG) was also normal. MRI discovered bilateral hip, knee, and bilateral suprapatellar bursa effusion (Figure 1).

Upon admission, IVIG-resistant KD was confirmed. The arthritis was believed to be associated with KD. We prescribed her a second dose of IVIG (2 g/Kg/day) and a high dose of oral aspirin (30 mg/kg/day). She was also given mannitol for suspected aseptic meningitis. Her rashes were fading away, and fever was reduced to around 38.5. However, her pain in both legs and fingers persisted. She was then given prednisone 2 mg/kg/day for two weeks. After this regimen, her temperature returned to normal, and the pain in legs and fingers backed down slowly. Aspirin sequentially reduced to a low dose (3 mg/Kg/day). At this stage, periungual desquamation started to emerge. The follow-up MRI revealed significantly reduced effusion. She became fully functional in mobilizing her extremities. The follow-up echocardiogram revealed progressively dilated coronary arteries involving the right coronary artery and the left main stem coronary artery (LMCA 2.7 mm, Z score = 1.73; RCA 2.3 mm, Z score = 1.56). She was discharged on prednisone 1 mg/kg/day and instructed to taper off over two weeks and continue low dose aspirin for 6 months. Postadmission ECHO follow-up showed gradual resolution of the coronary artery lesion. Echocardiogram revealed LMCA 2.2 mm (Z score = 0.37) and RCA 1.9 mm (Z score = 0.32). The patient was generally in good condition with the treatment regimen.

### 2.2. Case Two

A 3-year-old Chinese boy was admitted with a chief complaint of fever and neck swelling for four days. He also had intermittent abdominal pain and vomiting. Cefuroxime sodium was given intravenously for 2 days. His symptoms were not relieved. On examination, he was febrile (39.0) and tachycardic (140 beats/min). He had rashes covering most of his body surface. Multiple palpable cervical lymph nodes were tender to touch. He had pin eyes with clear discharge (nonpurulent conjunctivitis), red cracked lips and strawberry tongue, and pharyngeal erythema without exudate. On physical examination, he had no respiratory and cardiac abnormality. He had redness and swelling in his palms and soles.

On laboratory investigation, he had an elevated WCC of 13.38 × 10^9^/L (reference range: 3.5–9.5 × 10^9^/L), slightly decreased haemoglobin of 112 g/L (reference range: 120–140 g/L), and elevated platelets count of 491 × 10^9^/L (reference range: 100–300 × 10^9^/L). On the comprehensive metabolic panel (CMP), he had lower albumin level at 29 g/L (reference range: 40–55 g/L) and serum sodium 130.9 mmol/L (reference range: 135–145 mmol/L) and normal electrolytes, calcium, magnesium, and phosphate. The inflammatory marker tests showed CRP at 62.07 mg/L (reference range: 0–10 mg/L), ESR at 64 mm/h (reference range: 0–20 mm/h), and IL-6 at 80.03 pg/mL (reference range: ≤5.4 pg/mL). On lumbar puncture (LP), CSFP was 203 mmH_2_O (reference range: 80–180 mmH_2_O), CSF nucleated cell counts of 102 × 10^6^/L was elevated (reference range: 0–8 × 10^6^/L), and his cardiac focused exams did not find abnormality: BNP normal, echocardiogram normal, LMCA 2.6 mm (Z score = 1.21), RCA 2.1 mm (Z score = 0.7), and the standard 12-lead ECG normal.

He was admitted for classic KD. We started a high dose of IVIG (2 g/Kg/day) and aspirin (30 mg/kg/day). Mannitol was also given for suspected aseptic meningitis. After initial treatment, his skin rash and conjunctival congestion were largely improved but fever persisted. We gave a second dose of IVIG (2 g/Kg), and his fever stated to recede to no more than 37.8°C. On the 6th day of admission, he developed pain in the back, neck, legs, and fingers. The swelling in his extremities were evident, dominant in the left leg. His neck motion was limited by pain. He had difficulty in standing up or using his fingers to grip objects due to unbearable pain.

**Figure 2:**
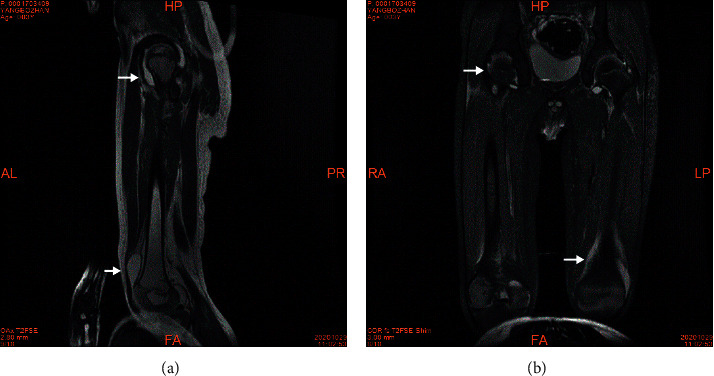
T2-weighted imaging in the acute phase of Kawasaki disease. There was a long T2 signal in the bilateral hips, knees, and bursae suprapatellaris (a), (b), which indicated arthritis with effusion, while there was an inflammatory edema signal in thigh skeletal muscle.

The repeating lab work showed increased WBC 22.95 × 10^9^/L, decreased haemoglobin 103 g/L, and increased CRP level 88.98 mg/L. Other lab findings were negative for anti-*Streptococcus* “O” antibody and negative detection for antibodies against RF, ANA, ds-DNA, Sm, SS-A, SS-B, U1RNP, RO-52, SCL-70, and PM-Scl. On image study, MRI revealed swelling in the skeletal muscle of the thigh, bilateral hip, knee, and suprapatellar bursa effusion (Figure 2). The aforementioned findings were consistent with arthritis that was likely associated with KD. We started prednisone (2 mg/kg/day) for two weeks. His low-grade fever was resolved, and the pain and swelling in the extremities were gradually alleviated. Aspirin was subsequently reduced to a low dose (3 mg/Kg/day). The follow-up MRI showed remarkably reduced effusion. Similar to the first case, the boy also had periungual desquamation, the signature skin manifestation in KD. He was discharged on prednisone 1 mg/kg/day and was instructed to taper off within two weeks. Aspirin was continued at a low dose for three months. The follow-up MRI showed completely resolved joint effusion. The echocardiogram showed no abnormality throughout the course. The patient had no complaints.

## 3. Discussion

According to the 6th edition diagnostic guideline of Kawasaki disease in Japan, Classic KD is diagnosed based on the presence of 6 principal clinical features [3]: fever; bilateral bulbar conjunctival injection; changes of the lips and oral cavity: reddening of lips, strawberry tongue, and diffuse injection of oral and pharyngeal mucosa; rash (including redness at the site of Bacille Calmette–Guerin (BCG) inoculation); changes of peripheral extremities: (initial stage) reddening of palms and soles and edema and (convalescent stage) periungual desquamation; and nonsupparative cervical lymphadenopathy. Approximately 10% to 20% of patients with KD develop recrudescent or persistent fever at least 36 hours after the end of their IVIG infusion and are termed IVIG resistant [2]. Our two cases were diagnosed as KD based on clinical features of fever, bilateral nonpurulent conjunctivitis, cervical lymphadenopathy, rash, red cracked lips, strawberry tongue, and diffuse erythema of the oropharynx, red palm and soles, and edema. They met the diagnostic criteria of complete KD. After initial treatment with IVIG and aspirin, fever continued. Therefore, they were defined as IVIG-resistant KD. The periungual desquamation also supported the diagnosis.

Severe arthritis with effusion is most commonly seen in children with SOJIA. Dong et al. reported that the incidence of SOJIA after treatment for presumed KD is 0.2% [4]. The two cases in this report shared similar spectrum of symptoms of KD and SOJIA. It is critical to determine whether the arthritis is secondary to KD or SOJIA. We favored diagnosis of KD based on the following features: (1) fever and rash in KD is typically a continuous process whereas the intermittent flares are more common in SoJIA and (2) sick appearance in KD occurs even in the afebrile stage whereas it is lacking in SoJIA if patients have no fever [5]. Based on clinical presentations in these two children and their response to the treatment, we believed the correct diagnosis was KD with arthritis. In addition, we considered other differential diagnosis and excluded them by disease-specific lab tastings: anti-*Streptococcus* “O” antibody was negative; blood cultures were negative; and antibody detection was negative for antibodies against RF, ANA, ds-DNA, Sm, SS-A, SS-B, U1RNP, RO-52, SCL-70, and PM-Scl.

IVIG-resistant KD is also known as refractory KD. Its treatment has been a controversial topic. The mainstream recommendations include [2] (1) a second dose of IVIG (2 g/kg); (2) high-dose steroids (usually methylprednisolone 20–30 mg/kg intravenously for 3 days, with or without oral prednisone tapering; and (3) a longer (eg, 2–3 weeks) tapering period of prednisolone or prednisone, together with IVIG 2 g/kg and aspirin or infliximab (5 mg/kg), etc. Following the recommendations, we prescribed a second dose of IVIG (2 g/kg) to treat these two patients. However, the fever was not well controlled. Rather, low-dose prednisone managed the symptoms well. It has been reported that by comparing with a second IVIG infusion, infliximab and intravenous methylprednisolone (IVMP) were more effective in terms of antipyretic effects [6]. We propose here that, for the IVIG-resistant KD, longer period of low-dose corticosteroids may be a better treatment option instead of a second dose of IVIG.

Kawasaki-disease-related arthritis has two types: early onset (<10th day of illness) with polyarticular disease and late-onset arthritis with oligoarticular involvement [7, 8]. The two cases in this report were early-onset type. To our knowledge, there is no standard treatment for KD-associated arthritis. Guleria et al. [1] reported that, among 40 cases diagnosed as KD-related arthritis, 8 (20%) children did not require any specific therapy for arthritis and 32 children (80%) were treated with nonsteroidal anti-inflammatory drugs (naproxen in 29; ibuprofen in 3). Among these patients, one case developed oligoarthritis in the acute phase of IVIF-resistant KD. His symptoms improved after infliximab and naproxen (15 mg/kg/day) treatment. However, he later developed polyarthritis at the convalescence phase, and the symptoms lasted for 6 months. His treatment included oral naproxen (15 mg/kg/day) for 2 months and prednisolone (initially, 2 mg/kg/day followed by tapering over 6 months). Manlongat and Allen [9] reported a 2-year-old boy with incomplete KD. His arthritis presented with bilateral hip synovitis and joint effusion. He was given IVIG and aspirin, followed by IV pulse methylprednisolone for three days. He was discharged on prednisolone. Takuma [10] reported a case of KD-related arthritis with synovial involvement. Cyclosporine A (CsA) was used for treatment. Burns [11] reported a successful infliximab treatment for IVIG-resistant KD in 16 KD with arthritis patients.

In this report, two pediatric patients with IVIG-resistant KD with arthritis had persistent joint pain even after a second dose of IVIG. The symptoms were relieved after four weeks of prednisone tapering. Other options for KD-associated arthritis have pros and cons: nonsteroidal anti-inflammatory drugs can relieve the joint symptoms but have no effect on fever; high-dose glucocorticoid and CsA have undesired side effects; and infliximab is not cost efficient. By comparison, low-dose prednisone might be a favorable treatment option for IVIG-resistant KD with severe arthritis.

## 4. Conclusions

There is no standardized treatment or guideline for IVIG-resistant KD combined with severe arthritis. We present two cases of IVIG-resistant Kawasaki disease with severe arthritis and joint effusion, and they benefited from low-dose prednisone finally. More research is expected to give us more appropriate treatment guidance.

## Data Availability

The data that support the findings of this study are available on request from all authors.

## References

[1] Guleria S., Pilania R. K., Jindal A. K. (2020). Clinico-laboratory profile of Kawasaki disease with arthritis in children. *European Journal of Pediatrics*.

[2] McCrindle B. W., Rowley A. H., Newburger J. W. (2017). Diagnosis, treatment, and long-term management of Kawasaki disease: a scientific statement for health professionals from the American heart association. *Circulation*.

[3] Kobayashi T., Ayusawa M., Suzuki H. (2020). Revision of diagnostic guidelines for Kawasaki disease (6th revised edition). *Pediatrics International*.

[4] Dong S., Bout-Tabaku S., Texter K., Jaggi P. (2015). Diagnosis of systemic-onset juvenile idiopathic arthritis after treatment for presumed Kawasaki disease. *The Journal of Pediatrics*.

[5] Sahin S., Adrovic A., Barut K., Kasapcopur O. (2017). Systemic-onset juvenile idiopathic arthritis or incomplete Kawasaki disease: a diagnostic challenge. *Clinical and Experimental Rheumatology*.

[6] Chan H., Chi H., You H. (2019). Indirect-comparison meta-analysis of treatment options for patients with refractory Kawasaki disease. *BMC Pediatrics*.

[7] Hicks R. V., Melish M. E. (1986). Kawasaki syndrome. *Pediatric Clinics of North America*.

[8] Gong G. W. K., McCrindle B. W., Ching J. C., Yeung R. S. M. (2006). Arthritis presenting during the acute phase of Kawasaki disease. *The Journal of Pediatrics*.

[9] Manlongat E. V., Allen W. C. (2017). Incomplete Kawasaki disease presenting as bilateral hip synovitis. *Journal of Paediatrics and Child Health*.

[10] Ito T., Hoshina T., Taku K., Kusuhara K. (2019). Kawasaki disease-related arthritis with synovial involvement. *Pediatrics International: Official Journal of the Japan Pediatric Society*.

[11] Burns J. C., Mason W. H., Hauger S. B. (2005). Infliximab treatment for refractory Kawasaki syndrome. *The Journal of Pediatrics*.

